# Plasma Lipidomic Remodeling in Behçet’s Disease Reveals Alterations Associated with Vascular Involvement

**DOI:** 10.3390/metabo16060363

**Published:** 2026-05-27

**Authors:** Engin Koçak, Çiğdem Yücel, Sevilay Erdoğan Kablan, Erdim Sertoğlu, Taner Özgürtaş, Emirhan Nemutlu, Ahmet Omma

**Affiliations:** 1 Department of Analytical Chemistry, Faculty of Gulhane Pharmacy, University of Health Sciences, Ankara 06018, Türkiye; 2 Department of Pharmaceutical Biochemistry, Faculty of Gulhane Pharmacy, University of Health Sciences, Ankara 06018, Türkiye; cigdem.yucel@sbu.edu.tr; 3 Department of Analytical Chemistry, Faculty of Pharmacy, Hacettepe University, Ankara 06100, Türkiye; sevilay.erdogan@hacettepe.edu.tr (S.E.K.); enemutlu@hacettepe.edu.tr (E.N.); 4 Department of Clinical Biochemistry, University of Health Sciences, Gulhane Training and Research Hospital, Ankara 06010, Türkiye; erdim.sertoglu@sbu.edu.tr (E.S.); taner.altuntas@sbu.edu.tr (T.Ö.); 5 Department of Rheumatology, Bilkent City Hospital, Ankara 06800, Türkiye; ahmetomma@gmail.com

**Keywords:** Behçet’s disease, vascular involvement, mass spectrometry, lipidomic profiling, biomarkers

## Abstract

**Highlights:**

**What are the main findings?**

- Plasma lipidomics revealed coordinated remodeling of membrane, signaling, and energy-related lipids in Behçet’s disease.
- Vascular involvement was associated with subtle, lipid-specific alterations rather than global lipidomic changes.

**What are the implications of the main findings?**

- Lipidomic alterations suggest integrated changes in membrane organization, inflammatory signaling, and metabolic adaptation in BD
- These findings provide a basis for future mechanistic and targeted lipidomic studies in vascular involvement.

**Abstract:**

**Background/Objectives**: Behçet’s disease (BD) is a chronic multisystem inflammatory disorder in which vascular involvement represents a major cause of morbidity and mortality. However, the molecular mechanisms underlying vascular involvement remain poorly understood. Lipidomics offers a powerful approach to investigate disease-associated metabolic alterations at the lipid level. **Methods**: Plasma lipidomic profiles were analyzed in 48 patients with BD, including 18 with vascular involvement, and 40 age- and sex-matched healthy controls. Lipids were extracted using a chloroform–methanol-based protocol and analyzed by LC-qTOF-MS. Data processing and lipid annotation were performed using MS-DIAL. Multivariate and univariate statistical analyses, together with class-based KEGG pathway mapping, were applied to evaluate lipidomic alterations. **Results**: Principal component analysis demonstrated a clear separation between BD patients and healthy controls, indicating extensive lipidomic remodeling. BD was associated with significant alterations in phosphatidylcholines, sphingomyelins, diacylglycerols, and triacylglycerols, reflecting coordinated changes in membrane structure, lipid-mediated signaling, and energy metabolism. Pathway analysis further supported the involvement of glycerophospholipid, glycerolipid, and phosphatidylinositol-related metabolic pathways. Comparison between BD patients with and without vascular involvement revealed no major global lipidomic shift; however, specific lipid species showed consistent alterations. Two phosphatidylcholine species (PC 33:2 and ether-linked PC 31:4e) were decreased, whereas one triacylglycerol species (TAG 58:2) was increased in patients with vascular involvement. **Conclusions**: These findings suggest that BD is characterized by coordinated lipidomic reprogramming involving membrane remodeling, inflammatory signaling, and metabolic adaptation. Vascular involvement appears to be associated with subtle, lipid-specific alterations rather than a global lipidomic shift, highlighting potential molecular features of disease progression.

## 1. Introduction

Behçet disease (BD) is a chronic, relapsing, multisystem inflammatory disorder characterized by recurrent oral aphthae, genital ulcers, ocular inflammation, and diverse involvement of the skin, joints, gastrointestinal system, and central nervous system. Classified among the variable-vessel vasculitides, BD uniquely affects arteries and veins of all sizes, resulting in substantial clinical heterogeneity [1,2]. Although its etiology remains incompletely understood, current evidence points to a multifactorial pathogenesis involving a strong genetic predisposition—particularly HLA-B*51—environmental triggers, and dysregulated innate and adaptive immune responses. Hallmark features include exaggerated neutrophil activation, endothelial dysfunction, and elevated pro-inflammatory cytokine activity. BD is most prevalent along the Silk Road and predominantly affects young adults, contributing significantly to long-term morbidity [1,2,3].

Vascular involvement is one of the most serious manifestations of BD. It could affect approximately 15–40% of patients and shows a clear male predominance [4]. Vascular Behçet (VBD) can affect arteries and veins of any size, though venous lesions—particularly deep vein thrombosis—are the most common, followed by arterial aneurysms, pseudoaneurysms, and occlusive arterial disease. Vascular complications typically emerge within the first several years following diagnosis and recur despite therapy [4,5]. Their pathogenesis is driven by exaggerated neutrophil activation, oxidative stress, and profound endothelial dysfunction, resulting in a markedly pro-thrombotic state independent of classical coagulation abnormalities [6]. These complications are major contributors to BD-related mortality, especially due to aneurysm rupture and pulmonary artery involvement.

Lipidomics has emerged as a powerful analytical and systems biology approach for characterizing global lipidome alterations and their roles in disease pathogenesis [7,8]. Although several studies have explored lipid remodeling in Behçet’s disease (BD), comprehensive evaluations of global lipidomic alterations remain limited. In particular, lipidomic alterations associated with vascular involvement in BD have not been systematically investigated. Previous lipidomics studies have demonstrated disturbances in phospholipid and oxylipin mediators in blood and immune cells, which may contribute to the regulation of vascular tone, coagulation, and inflammatory processes. Ben-Fradj et al. used a targeted lipidomics approach to analyze various oxylipin species in blood samples and suggested that altered oxylipin profiles may contribute to platelet dysfunction, hemostatic imbalance, thrombosis, and vascular dysregulation in BD [9]. In addition, Park et al. analyzed the metabolomic profile of peripheral blood mononuclear cells and reported that altered phospholipid metabolism may be associated with endothelial injury and thrombotic processes [10]. Nevertheless, previous lipidomics investigations were not specifically focused on vascular involvement in Behçet’s disease, and comprehensive characterization of vascular-associated lipidomic remodeling remains limited.

To address this gap, the present study aimed to characterize lipidomic remodeling in BD and to identify additional alterations associated with vascular involvement (VBD). Building on our previous metabolomics study, which demonstrated dynamic changes in the plasma polar metabolome in BD and additional modifications in VBD [11], we performed an in-depth lipidomic analysis to provide a more comprehensive understanding of disease-related metabolic alterations.

## 2. Materials and Methods

### 2.1. The Study Population

This study employed a cross-sectional design and enrolled 48 individuals diagnosed with BD, including 18 patients presenting with vascular involvement (VBD), alongside 40 healthy volunteers matched for age and sex and recruited from routine rheumatology outpatient visits. Participants were excluded if they had any concurrent autoimmune or inflammatory disorders, active or recent infections, anemia, chronic metabolic conditions, cardiovascular disease, impaired renal or hepatic function, thyroid dysfunction, malignancy, or if they were pregnant or within six months postpartum [11].

Ethical approval for the study was obtained from the Ethics Committee of Gülhane Training and Research Hospital (Decision No. 2020/15). All procedures were conducted in accordance with the principles of the Declaration of Helsinki (2000 revision), and written informed consent was obtained from all participants prior to enrollment.

The diagnosis of BD was established according to the criteria of the International Study Group. Demographic information and relevant clinical characteristics were systematically recorded [12]. Disease activity was assessed using the validated Turkish version of the Behçet Disease Current Activity Form (BDCAF), which evaluates clinical manifestations such as headache, oral and genital ulceration, erythema nodosum, papulopustular lesions, arthralgia or arthritis, and gastrointestinal, ocular, neurological, and major vascular involvement [13]. The BDCAF score was calculated by summing the presence of each item, yielding a total score ranging from 0 to 12. Patients classified as having vascular involvement were those who exhibited active vasculitic lesions at the time of sample collection, including deep vein thrombosis, arterial plaques, stenosis, occlusions, or aneurysms.

The demographic and clinical characteristics of BD patients, including disease activity, BMI, vascular involvement status, and treatment information, are summarized in Table 1. Patients with vascular involvement had active vasculitic vascular lesions during sample collection. Treatment regimens were broadly comparable between BD and VBD groups and mainly consisted of corticosteroids, colchicine, and immunosuppressive agents. Blood samples were collected under overnight fasting conditions prior to plasma isolation and lipidomic analysis.

### 2.2. Plasma Lipidomics Analysis

Plasma samples were stored at −80 °C until analysis, and freeze–thaw cycles were minimized prior to lipid extraction. Sample preparation, LC–MS acquisition, and data processing procedures were performed based on our previously established untargeted lipidomics workflow [14].

A chloroform:methanol:water co-solvent system (3:4:3, *v*/*v*/*v*) was used to separate lipids from other biomolecules. A quantity of 100 µL of plasma was mixed with 400 µL of ice-cold methanol (Sigma, St. Louis, MO, USA), followed by the addition of 300 µL chloroform (Sigma, St. Louis, MO, USA), and 300 µL water to achieve the final solvent ratio. Lipid species were collected from the lower chloroform (organic) phase. After isolation, the chloroform phase was evaporated overnight at 4 °C using a vacuum concentrator. The dried lipid extracts were reconstituted in 300 µL of isopropanol/acetonitrile (Sigma, St. Louis, MO, USA), (7:3, *v*/*v*), vortex-mixed, and filtered prior to LC–MS analysis [15]. All samples were processed using the same standardized extraction workflow under identical experimental conditions to minimize technical variability during lipid extraction and sample preparation.

Lipidomic profiling was performed using an Agilent 6530 LC-qTOF-MS system (Agilent Technologies, Santa Clara, CA, USA). Chromatographic separation was achieved on a Poroshell HPH C18 column (100 × 2.1 mm, 2.7 µm) using a binary solvent system consisting of water/acetonitrile (6:4, *v*/*v*) containing 0.1% formic acid and 10 mM ammonium formate (mobile phase A), and isopropanol/acetonitrile (9:1, *v*/*v*) supplemented with 0.1% formic acid and 10 mM ammonium formate (mobile phase B). The flow rate was maintained at 0.25 mL/min, and the column temperature was set to 60 °C.

Mass spectrometric data were acquired in both positive and negative electrospray ionization modes over an *m*/*z* range of 100–1700, using a medium isolation width. Tandem MS (MS/MS) experiments were performed using stepped collision energies of 10, 20, and 40 V. Quality control (QC) samples were prepared by pooling aliquots from all study samples and were analyzed throughout the analytical sequence following the same workflow to monitor instrument performance and ensure extraction and analytical reproducibility.

Raw LC–MS/MS data were processed using MS-DIAL software (version 4.92) for peak detection, deconvolution, alignment, gap filling, and normalization. Lipid species were putatively annotated based on MS/MS spectra acquired at 10, 20, and 40 V, using a 70% spectral similarity threshold against the MS-DIAL LipidBlast library. Signal intensity normalization was performed during MS-DIAL processing to minimize technical variability across samples. Pooled QC samples were analyzed throughout the analytical sequence to monitor extraction and analytical reproducibility and instrument stability. Only lipid features meeting QC reproducibility criteria (CV < 30%) were retained for downstream statistical analysis.

### 2.3. Statistical Analysis

The MetaboAnalyst 6.0 platform was used for statistical analysis. Principal component analysis (PCA) was performed to assess global lipidome structure and clustering trends among groups. Moreover, the tight clustering of pooled QC samples in PCA analysis further supported analytical stability and reproducibility during data acquisition.

In addition to principal component analysis (PCA), supervised multivariate analysis was performed using partial least squares discriminant analysis (PLS-DA). Model robustness was evaluated using cross-validation and permutation testing. Variable importance in projection (VIP) scores were used to identify lipid species contributing to group separation.

Univariate statistical analysis was conducted using a Student’s *t*-test with false discovery rate (FDR) correction (*p* < 0.05) and a fold-change threshold of ≥2 to identify significantly altered lipid species between experimental groups.

### 2.4. Class-Based Pathway Analysis

Due to limited compound-level KEGG annotations for individual lipid species, pathway analysis was performed using a lipid class–based mapping approach. Significantly altered lipid species were first grouped according to their lipid classes. Each lipid class was then assigned to relevant KEGG metabolic pathways.

## 3. Results

**Figure 1:**
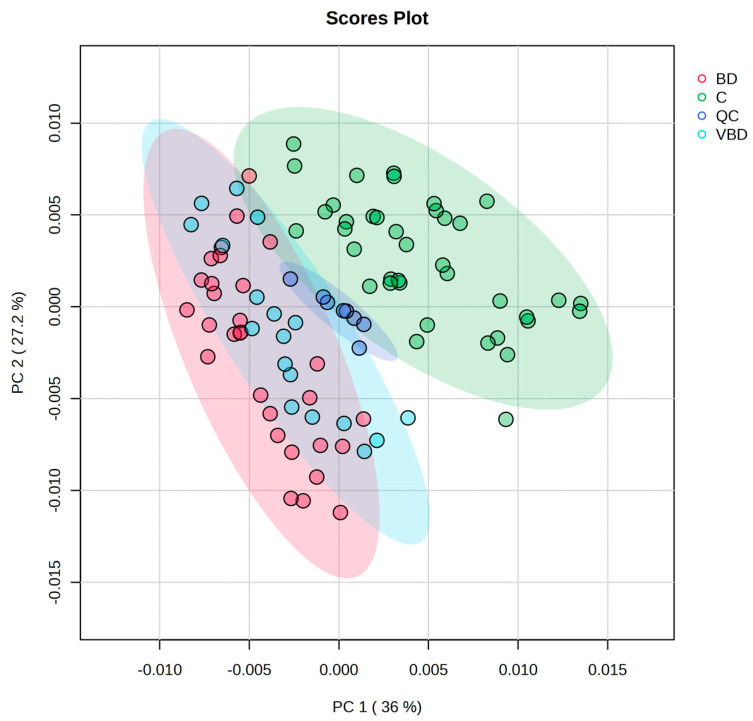
Principal component analysis of Behcet (BD), healthy control (C), vascular involvement in Behçet’s disease (VBD), quality control samples (QC) (F-value: 33.085, R^2^: 0.4462, *p*-value (based on 999 permutations): 0.01).

In the present work, we focused on lipid remodeling in Behçet’s disease (BD) and vascular involvement associated with BD. An LC–MS-based lipidomics approach was used to analyze the overall plasma lipidome profiles of healthy controls, BD, and vascular Behçet’s disease (VBD) groups. Multivariate analyses were performed to evaluate global lipidomic variation among groups. PCA analysis showed distinct clustering trends between healthy controls and both BD and VBD groups, suggesting disease-associated alterations in lipidomic structure (Figure 1). Consistently, PERMANOVA analysis confirmed that the control group was significantly distinct from both BD and VBD, whereas no significant difference was detected between BD and VBD groups (Supplementary Materials Table S1). Additional supervised multivariate analysis using partial least squares discriminant analysis (PLS-DA) further supported lipidomic differences between BD patients and healthy controls. Cross-validation analysis demonstrated strong model performance (R^2^ = 0.85, Q^2^ = 0.83), while permutation testing indicated that the observed separation was unlikely to occur by chance (*p* < 0.001). In contrast, PLS-DA analysis comparing BD and VBD groups demonstrated only modest clustering trends with partial overlap and limited predictive performance (Q^2^ ≈ 0.33), supporting the interpretation that vascular involvement is associated with relatively subtle lipidomic alterations rather than extensive global lipidomic remodeling (Supplementary Materials Figures S1 and S2). Quality control (QC) samples formed a tight cluster, indicating good analytical reproducibility and instrument stability throughout the LC–MS analysis. Moreover, the majority of detected lipid species (84.6%) exhibited QC coefficient of variation (CV) values below 30%, indicating acceptable analytical reproducibility and stability of the LC–MS platform.

### 3.1. Comparison of Lipid Profile Between BD and Healthy Control

**Figure 2:**
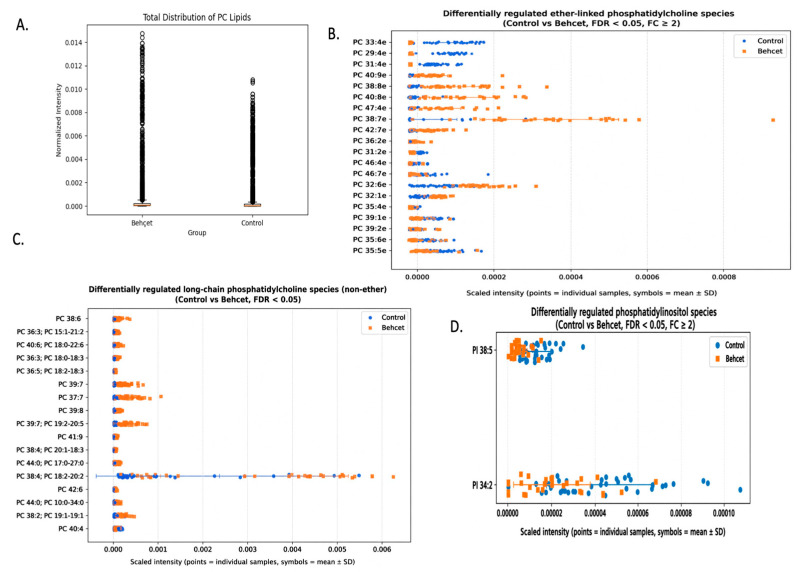
(**A**) Total distribution of PC lipids in BD and control groups; (**B**) Statistically altered ether-linked PC species between BD and control groups; (**C**) Statistically altered long-chain PC species (non-ether) between BD and C groups; (**D**) Statistically altered PI lipids between BD and C groups. (BD: Behçet’ s disease, C: healthy control, FDR adjusted *p* < 0.05, FC cut off ≥ 2).

In the second step, we focused on comparison of BD and healthy control to evaluate systematic lipidomic alterations. Results showed that the phosphatidylcholine (PC) profile was significantly altered with BD progression. We observed that the overall distribution of PC species showed more variability in BD groups (Figure 2A). We also observed that various ether-linked PC species, which are multifunctional lipids, were altered significantly between BD and healthy groups (Figure 2B). The long-chain phosphatidylcholine (PC) profile showed an increasing trend in BD groups (Figure 2C). Two phosphatidylinositols (PI) species were decreased in BD groups (Figure 2D).

**Figure 3:**
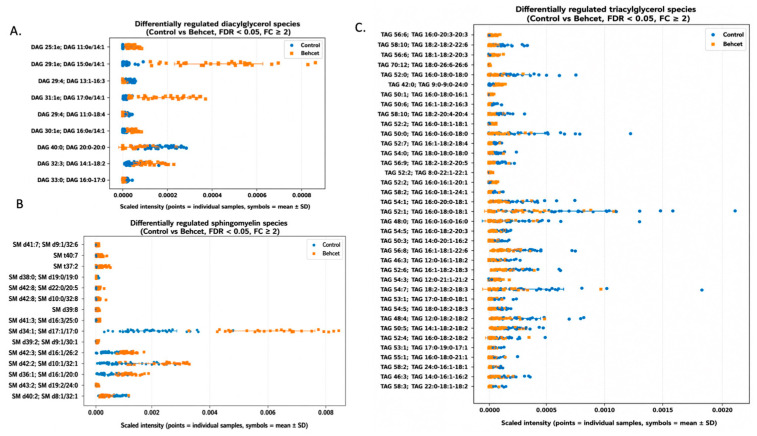
(**A**) Statistically altered DAG lipids between BD and C groups; (**B**) Statistically altered TAG species between BD and C groups; (**C**) Significantly altered sphingomyelins (SM) species between BD and control groups. (BD: Behçet’ s disease, C: control, FDR adjusted *p* < 0.05, FC cut off ≥ 2).

We examined the diacylglycerols (DAGs) profile based on PI results and the results showed that there is a dysregulation in the DAGs profile (Figure 3A). Another important point is differentiation of the sphingomyelins profile, which is the major component of sphingolipids (Figure 3B). We also observed that the triacylglycerol (TGA) profile was changed in BD. These findings may provide additional insight into BD-associated lipid remodeling (Figure 3C).

**Figure 4:**
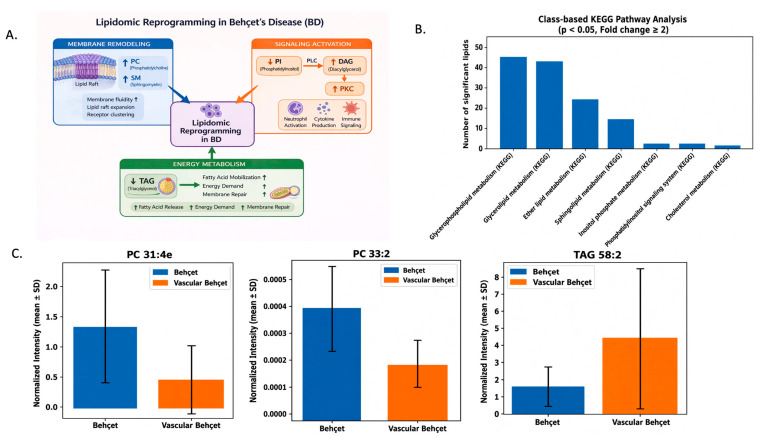
(**A**) Mechanistic integration of lipidomic alterations in Behçet disease. Coordinated changes in phosphatidylcholines (PC), sphingomyelins (SM), phosphatidylinositol (PI), diacylglycerols (DAG), and triacylglycerols (TAG) suggest potential membrane remodeling, activation of PI–PLC–DAG inflammatory signaling, oxidative stress, and energy mobilization in BD compared with controls. (**B**) Class-based pathway analysis for altered lipids. (**C**) Statistically altered lipids in VBD. (FDR adjusted *p* < 0.05, FC cut off ≥ 2).

We used altered lipids to evaluate the mechanistic basis of BD at lipid level (Figure 4A). Moreover, we also used significantly altered lipids in class-based pathway analysis. The results showed that various biological processes were altered with BD progression (Figure 4B).

### 3.2. Comparison of Lipid Profile Between BD and VBD Groups

We further explored lipidomic alterations associated with vascular involvement in Behçet’s disease by comparing BD and VBD groups. PCA analysis did not demonstrate a distinct global lipidomic separation between the two groups. Although supervised PLS-DA analysis showed partial group discrimination, the separation pattern was substantially weaker compared with the BD versus control comparison, supporting the presence of relatively limited lipidomic alterations associated with vascular involvement. Consistent with this observation, only a limited number of lipid species were significantly altered in VBD patients compared with BD patients. Specifically, two phosphatidylcholine species (PC 33:2 [PC 15:0–18:2] and ether-linked PC 31:4e) were decreased, whereas one triacylglycerol species (TAG 58:2 [TAG 16:0–24:0–18:2]) was increased in VBD compared with BD (Figure 4C). These findings suggest that vascular involvement in BD may be associated with relatively subtle and selective lipidomic alterations rather than broad global lipid remodeling. However, these observations should be interpreted cautiously and require validation in larger independent cohorts.

## 4. Discussion

Multivariate analysis using principal component analysis (PCA) revealed a clear separation among healthy controls (C), Behçet’s disease (BD), and vascular Behçet’s disease (VBD) groups (Figure 1). The PCA results indicated a pronounced shift in the global lipid profile in BD compared with controls. In contrast, no marked separation was observed between BD and VBD, suggesting that vascular involvement is associated with alterations in specific lipid species rather than a global lipidomic reorganization.

### 4.1. Differentiation of Lipid Profile Between BD and Healthy Control

In the present study, we first investigated lipidomic alterations associated with Behçet’s disease (BD) in the absence of vascular involvement. Since vascular manifestations typically develop within 1–5 years following initial diagnosis, the primary focus was placed on characterizing global lipidomic remodeling in BD. Subsequently, additional lipidomic changes associated with vascular involvement (VBD) were evaluated.

Lipidomic analysis revealed extensive remodeling of the plasma lipid profile in BD compared with healthy controls. Statistical analysis demonstrated that multiple lipid classes were significantly altered in BD (Supplementary Materials Table S2).

#### 4.1.1. Phosphatidylcholines (PCs)

Lipidomic analysis revealed substantial alteration in the phosphatidylcholine (PC) profile in BD compared with healthy controls, with 36 PC species increased and 11 decreased (Supplementary Materials). PCs are major structural components of cellular membranes and play essential roles in lipoprotein metabolism, membrane fluidity, and inflammatory signaling [16].

The overall distribution of PC species exhibited greater variability in BD patients, with an expanded upper range of intensities compared with controls (Figure 2A). This pattern suggests altered PC homeostasis potentially associated with inflammatory processes in BD. Consistent with our findings, Zheng et al. also reported significant alterations in serum PC profiles in BD patients relative to healthy controls [17].

The increased variability observed in several phosphatidylcholine species among BD patients may reflect the marked clinical and immunological heterogeneity characteristic of Behçet’s disease. Differences in inflammatory burden, endothelial dysfunction, oxidative stress, vascular involvement status, disease activity, and treatment exposure may contribute to inter-individual variation in phospholipid remodeling. Given the central structural and signaling roles of phosphatidylcholines in membrane dynamics and inflammatory regulation, altered variability patterns may represent heterogeneous metabolic responses associated with disease-related vascular and inflammatory processes. However, these observations remain exploratory and should be validated in larger longitudinal studies.

We further observed significant alterations in ether-linked phosphatidylcholine (PC) species in BD compared with controls (Figure 2B). Ether-linked PCs are functionally important lipids involved in membrane dynamics, contributing to membrane fluidity, lipid raft organization, and protection of polyunsaturated fatty acids against oxidative damage due to their characteristic vinyl ether bond structure [18]. Dysregulation of ether-linked lipids has been previously associated with inflammatory and autoimmune conditions and is considered indicative of increased oxidative stress. In line with this, Park et al. reported alterations in ether-linked lysophosphatidylethanolamines in peripheral blood mononuclear cells from BD patients [10]. Taken together, the observed changes in ether-linked PC species may be associated with oxidative stress-related lipid remodeling in BD. These findings may highlight ether-linked PC remodeling as a potential lipidomic feature associated with oxidative stress in BD.

We observed a general increasing trend in long-chain phosphatidylcholine (PC) species in BD compared with controls (Figure 2C). Long-chain, particularly polyunsaturated PC species, play critical roles in regulating membrane fluidity, curvature, and lipid raft organization, and are closely involved in inflammatory signaling processes [19].

The elevation of long-chain PC species may reflect adaptive membrane remodeling responses aimed at preserving membrane integrity under conditions of chronic inflammation [20]. Collectively, these alterations may reflect enhanced membrane remodeling and increased oxidative and inflammatory stress in BD.

#### 4.1.2. Phosphatidylinositols (PIs)

Phosphatidylinositols (PIs) are key membrane phospholipids that serve as precursors for multiple second messengers and play central roles in intracellular signaling and inflammatory responses. In the present study, two PI species were significantly decreased in BD compared with healthy controls (Figure 2D).

Although direct evidence regarding PI alterations in BD is limited, previous proteomic studies have highlighted the involvement of phosphoinositide-related signaling components, including phosphoinositide-3-kinase adaptor protein 1 (PIK3AP1), in disease pathogenesis [21]. These observations suggest that PI-mediated signaling pathways, particularly those related to PI3K–phosphoinositide signaling, may be dysregulated in BD. In this context, the observed reduction in PI species may reflect alterations in upstream lipid signaling pathways that regulate immune activation and inflammatory responses. Given that phosphatidylinositols serve as precursors for diacylglycerol (DAG), these alterations may also indicate downstream effects on PI–PLC–DAG signaling pathways involved in inflammatory regulation.

#### 4.1.3. Diacylglycerols (DAGs)

Diacylglycerols (DAGs) are key lipid-derived second messengers that activate protein kinase C (PKC), thereby regulating neutrophil activation, cell adhesion, and cytokine production [22,23]. In the present study, multiple DAG species were significantly altered in BD compared with controls (Figure 3A). Although direct evidence regarding DAG-specific alterations in BD is limited, the concurrent decrease in phosphatidylinositol (PI) species observed in this study suggests a potential shift in upstream phospholipase C (PLC)-mediated lipid signaling. Given that PIs serve as precursors for DAG generation, these combined alterations may indicate modulation of the PI–PLC–DAG signaling axis. This pathway is known to regulate key inflammatory processes, and its alteration may contribute to enhanced immune activation and inflammatory responses in BD [22,24].

#### 4.1.4. Sphingomyelins (SMs)

Sphingomyelins (SMs) are major sphingolipid components of lipoproteins and cellular membranes, playing important roles in membrane organization and signaling. In the present study, the majority of significantly altered SM species were increased in BD compared with healthy controls (Figure 3B).

Alterations in SM metabolism have been widely associated with metabolic and inflammatory conditions. Increased SM levels may promote lipid raft expansion, facilitating cytokine receptor clustering and amplification of inflammatory signaling pathways [25,26]. In this context, the observed elevation of SM species may reflect membrane reorganization and contribute to endothelial dysfunction and sustained inflammatory responses in BD.

#### 4.1.5. Triacylglycerols (TAGs)

Triacylglycerols (TAGs) are major circulating lipids transported within lipoproteins and serve as key reservoirs for energy storage and distribution. In the present study, a global decrease in TAG species was observed in BD compared with healthy controls (Figure 3C). Reduced TAG levels may indicate increased fatty acid mobilization and elevated energy demand associated with chronic inflammatory conditions. In addition, enhanced lipid utilization may reflect metabolic adaptations linked to membrane remodeling and repair processes [27].

#### 4.1.6. Biological Implications of Altered Lipid Classes

Compared with controls, BD exhibits pronounced remodeling of membrane-associated lipids, characterized by coordinated changes in long-chain, polyunsaturated, and ether-linked phosphatidylcholines, together with widespread alterations in sphingomyelin species. These changes suggest a reorganization of membrane architecture and lipid raft domains (Figure 4A).

Concurrently, selective modulation of phosphatidylinositols and diacylglycerols points to alterations in PI–PLC–DAG signaling pathways involved in inflammatory and immune responses. The combined changes in these lipid classes indicate a coordinated shift in lipid-mediated signaling processes.

In addition, the observed alterations in diacylglycerols and triacylglycerols suggest enhanced lipid mobilization and redistribution of energy resources under chronic inflammatory conditions.

#### 4.1.7. Class-Based Pathway Analysis of Altered Lipids 

In the present study, a lipidomics-driven, mechanistic approach was applied to better understand the molecular basis of BD pathogenesis. Due to the limited availability of compound-level pathway annotations for individual lipid species, a class-based KEGG pathway analysis was performed using significantly altered lipid groups.

This analysis revealed predominant involvement of glycerophospholipid and glycerolipid metabolism (Figure 4B), indicating widespread remodeling of membrane-associated and energy-related lipid pathways. Consistent with these findings, previous studies have shown that dysregulated glycerophospholipid metabolism may contribute to monocyte dysfunction in BD [28].

In addition, alterations in inositol phosphate and phosphatidylinositol metabolism further support the involvement of PI-derived second messenger systems. These pathways play central roles in regulating immune cell activation, endothelial function, and inflammatory signaling, suggesting that lipid-mediated signaling dysregulation is an integral component of BD pathophysiology.

The mechanistic interpretations presented in this study are based on exploratory untargeted lipidomics analyses and pathway-level associations. Therefore, these findings should be considered hypothesis-generating observations requiring further functional and targeted validation studies.

### 4.2. Lipid-Based Differentiation with Vascular Involvement in BD

In the present study, lipidomic profiles of patients with vascular involvement (VBD) were compared with those without vascular manifestations (BD). Vascular complications in BD typically develop within the first 1–5 years following diagnosis; however, the molecular mechanisms underlying this progression remain poorly understood. To date, most lipidomics studies have focused on BD in general, with limited attention to vascular involvement.

In our analysis, no dramatic differences were observed in the global lipidomic profile between BD and VBD, indicating that vascular involvement is not associated with a large-scale lipidomic shift. Instead, a limited number of lipid species showed consistent alterations. Specifically, two phosphatidylcholine species (PC 33:2 (PC 15:0–18:2) and ether-linked PC 31:4e) were decreased, whereas one triacylglycerol species (TAG 58:2 (TAG 16:0–24:0–18:2)) was increased in VBD compared with BD (Figure 4C).

These findings suggest that vascular involvement in BD may be associated with subtle lipid-specific alterations rather than extensive global lipidomic remodeling. The observed reductions in PC and ether-linked PC species may be associated with oxidative-stress-related lipid and membrane remodeling, while the increase in TAG species may indicate altered energy utilization. Together, these results may highlight subtle but potentially meaningful lipid alterations associated with vascular involvement, which may reflect localized metabolic adaptations rather than global lipidomic reprogramming.

### 4.3. Limitations

This study has several limitations that should be considered when interpreting the findings. First, the sample size of the vascular Behçet’s disease (VBD) subgroup was relatively limited (*n* = 18), which may have reduced the statistical power to detect more subtle lipidomic differences and limits the generalizability of the results. In addition, stable-isotope-labeled internal standards were not used in the present untargeted lipidomics workflow. Therefore, the reported lipid alterations should be interpreted as relative rather than absolute quantitative changes. Future targeted lipidomics studies using isotope-labeled standards are warranted to validate these findings.

## 5. Conclusions

In conclusion, this study demonstrates that Behçet’s disease is associated with substantial alterations in plasma lipid profiles involving membrane-associated, signaling-related, and energy-related lipid classes. These findings provide insight into potential lipidomic changes linked to BD pathophysiology. In addition, vascular involvement was associated with limited but consistent alterations in specific lipid species. Further functional and targeted studies are required to validate the biological significance of these observations.

## Figures and Tables

**Table 1:** Demographic and clinical characteristics of Behçet’s disease patients [11].

Parameter	Values
Age at diagnosis [years], mean ± SD	32.3 ± 4.9
Disease duration [months], median (IQR)	94.8 (71.7)
Body mass index (BMI), mean ± SD	23.3 ± 2.7
Active disease, *n* (%)	28 (58.3)
BDCAF, median (IQR)	2.0 (0.0–3.0)
Disease symptoms in patient population, *n* (%)	
Oral ulcers	48 (100)
Genital ulcers	36 (75)
Erythema nodosum	24 (50)
Papulopustular lesions	28 (58.3)
Positive pathergy test	12 (25)
Arthritis	21 (43.7)
Uveitis	24 (50)
**Vascular involvement:**	18 (37.5)
Venous thrombosis	16 (89)
Arterial aneurism	1 (5.5)
Pulmonary aneurism	1 (5.5)

## Data Availability

The data presented in this study are available from the corresponding author upon reasonable request.

## Supplementary Materials

The following supporting information can be downloaded at: https://www.mdpi.com/article/10.3390/metabo16060363/s1, Table S1: permonova analysis; Table S2: List of significantly altered lipid species; Figure S1: (A) PLS-DA analysis of C and BD groups (B) VIP score of lipids (C) permutation analysis of model (R^2^ = 0.85, Q^2^ = 0.83) (C: Healthy control groups, BD: Behcet’ s disease group); Figure S2: (A) PLS-DA analysis of BD and VBD groups (B) VIP score of lipids (C) permutation analysis of model (R^2^ = 0.80, Q^2^ = 0.33) (C: Healthy control groups, BD: Behcet’ s disease group).
