# Supplementary material for: Plasma Lipidomic Remodeling in Behçet’s Disease Reveals Alterations Associated with Vascular Involvement
Source: Metabolites. 2026 May 27;16(6):363. doi: 10.3390/metabo16060363 (PMC13302958; doi:10.3390/metabo16060363)
Supplement: Supplementary file 1 [file metabolites-16-00363-s001.zip › Supplementary information Figure s1 and S2docx.pdf]

Supplementary information S2

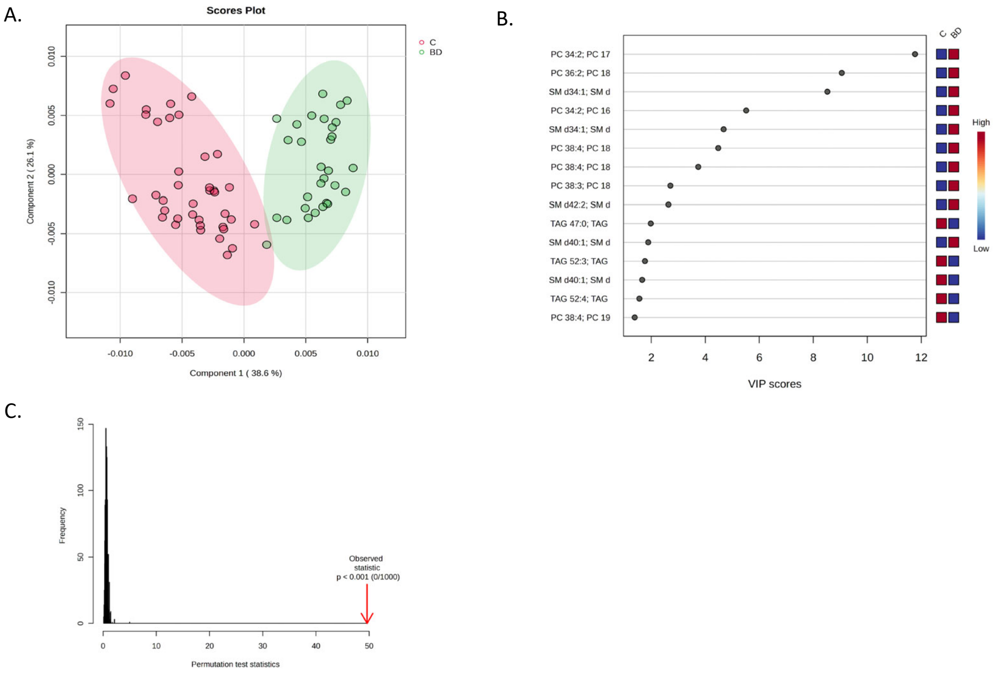

Figure S1. A) PLS-DA analysis of C and BD groups B) VIP score of lipids C) permutation analysis of model (R^2 = 0.85, Q^2 = 0.83) (C: Healthy control groups, BD: Behcet' s disease group)

Supplementary information S3

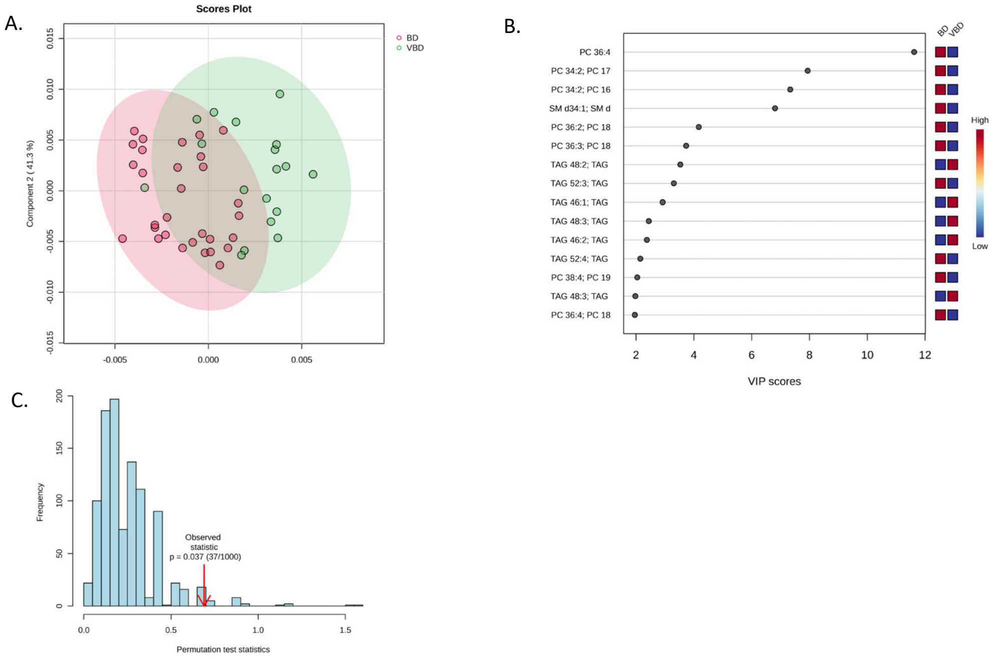

Figure S2. A) PLS-DA analysis of BD and VBD groups B) VIP score of lipids C) permutation analysis of model (R^2 = 0.80, Q^2 = 0.33) (C: Healthy control groups, BD: Behcet' s disease group)
